# Enhancing Alzheimer’s Diagnosis with Machine Learning on EEG: A Spectral Feature-Based Comparative Analysis

**DOI:** 10.3390/diagnostics15172190

**Published:** 2025-08-29

**Authors:** Yeliz Senkaya, Cetin Kurnaz, Ferdi Ozbilgin

**Affiliations:** 1 Department of Computer Applications, Akkus Vocational School, Ordu University, 52950 Ordu, Türkiye; yelizsenkaya@odu.edu.tr; 2 Department of Electrical and Electronics Engineering, Faculty of Engineering, Ondokuz Mayıs University, 55139 Samsun, Türkiye; 3 Department of Electrical and Electronic Engineering, Faculty of Engineering, Giresun University, 28200 Giresun, Türkiye; ferdi.ozbilgin@giresun.edu.tr

**Keywords:** Alzheimer’s disease (AD), EEG signal, machine learning (ML), feature extraction, support vector machines (SVMs), k-Nearest Neighbors (k-NN)

## Abstract

**Background/Objectives:** Alzheimer’s disease (AD) is a devastating neurodegenerative disorder that progressively impairs cognitive, neurological, and behavioral functions, severely affecting quality of life. The current diagnostic process relies on expert interpretation of extensive clinical assessments, often leading to delays that reduce the effectiveness of early interventions. Given the lack of a definitive cure, accelerating and improving diagnosis is critical to slowing disease progression. Electroencephalography (EEG), a widely used non-invasive technique, captures AD-related brain activity alterations, yet extracting meaningful features from EEG signals remains a significant challenge. This study introduces a machine learning (ML)-driven approach to enhance AD diagnosis using EEG data. **Methods:** EEG recordings from 36 AD patients, 23 Frontotemporal Dementia (FTD) patients, and 29 healthy individuals (HC) were analyzed. EEG signals were processed within the 0.5–45 Hz frequency range using the Welch method to compute the Power Spectral Density (PSD). From both the time-domain signals and the corresponding PSD, a total of 342 statistical and spectral features were extracted. The resulting feature set was then partitioned into training and test datasets while preserving the distribution of class labels. Feature selection was performed on the training set using Spearman and Pearson correlation analyses to identify the most informative features. To enhance classification performance, hyperparameter tuning was conducted using Bayesian optimization. Subsequently, classification was carried out using Support Vector Machines (SVMs) and k-Nearest Neighbors (k-NN) the optimized hyperparameters. **Results:** The SVM classifier achieved a notable accuracy of 96.01%, outperforming previously reported methods. **Conclusions:** These results demonstrate the potential of machine learning-based EEG analysis as an effective approach for the early diagnosis of Alzheimer’s Disease, enabling timely clinical intervention and ultimately contributing to improved patient outcomes.

## 1. Introduction

In light of technological advancements, there have been many developments in the field of healthcare, which, along with these advances, have increasingly prolonged human life by improving the diagnosis and treatment of diseases. With the increase in life expectancy, the global elderly population is also rapidly rising. This situation has particularly led to an increase in dementia, a health issue that commonly arises during aging. Dementia is a neurodegenerative disease characterized by declining cognitive and behavioral functions, especially memory, due to the death of brain cells (neurons) caused by aging or specific neurological conditions [1]. According to the “World Alzheimer Report” published in 2018, approximately 50 million people worldwide have dementia, and by 2050, the number of dementia cases is projected to exceed 152 million [2].

Alzheimer’s disease (AD) accounts for a significant proportion of dementia cases, comprising 60–70% [3]. This situation has made AD a global issue [4]. AD is an irreversible neurodegenerative disease characterized by a progressive loss of neurological, mental, and cognitive functions, including changes in emotions, behavior, memory, language, and judgment [5,6,7,8]. In individuals with AD, brain electrical activity slows down compared to healthy individuals, manifesting as impairments in cognitive functions [1]. When examining the age distribution of individuals with this disease, the prevalence of AD is 1% among people aged 60–64. In comparison, this rate rises to 38% in individuals over the age of 85, clearly indicating that AD increases with advancing age [9]. Individuals with this disease are diagnosed based on prolonged tests and examinations by experienced professionals, and the accuracy of these diagnoses ranges between 85% and 93% [10].

Although there is currently no existing cure for AD, it is believed that some medications can slow the progression and, consequently, the symptoms of the disease if the diagnosis is made as early as possible. Performing a rapid diagnosis of AD is crucial for effectively using these medications during their effective period, significantly impacting the progression of the disease. A prompt diagnosis allows for the commencement of treatment before permanent brain damage occurs and enables the treatment of potential psychiatric symptoms such as depression and psychosis. With accurate diagnosis and treatment, patients may have the opportunity to maintain their personal needs and care for longer. Additionally, an early diagnosis allows the patient’s relatives to gain information about the disease and make financial and emotional plans for future situations [11]. Considering all these factors, the importance of diagnosing AD becomes clearer for the patient, their relatives, and society as a whole.

EEG (electroencephalogram) signals are recordings of the brain’s electrical activity, captured through electrodes or transducers placed on the scalp. These signals reflect the complex neuronal dynamics associated with brain function [12].

The EEG was first introduced into the literature by Hans Berger in 1929 as a method for recording electrical activity in the human brain [13]. Since Hans Berger’s first observation of pathological EEG sessions in a patient with a confirmed diagnosis of AD [14], numerous studies have been conducted on AD using EEG signals. Particularly in the last 20 years, EEG has been employed as a useful tool for diagnosing dementia [15]. EEG signals are of great importance for diagnosing brain-related diseases. One of the major advantages of EEG signals is their ability to capture brain signals without surgical intervention. EEG signals are also more time- and cost-effective than other methods, significantly increasing their use in diagnosing AD.

When examining the EEG signals of patients with AD, certain abnormalities are detected compared to the EEG signals of healthy individuals. The most notable characteristic of these abnormalities is the slowing of rhythms and decreased coherence between different brain regions. There is an increase in theta and delta band activities and a decrease in alpha and beta band activities. Additionally, there is a decrease in coherence within the alpha and beta bands. These abnormalities increase the severity of the disease [15].

This study aims to classify EEG signals from individuals with Alzheimer’s Disease (AD), Frontotemporal Dementia (FTD), and healthy individuals (HC) using machine learning (ML) methods after signal processing stages. By facilitating the earliest possible diagnosis of AD and improving decision-making times for professionals, the goal is to enable patients to lead a more comfortable life for a longer period. In recent years, machine learning techniques have been increasingly employed in healthcare to facilitate early diagnosis and improve clinical outcomes for complex disorders [7,8,9,10,16,17,18]. As reported in existing studies, ML methods were chosen for classification due to their superior performance compared to traditional approaches. Additionally, it contributes to the growing body of literature on the use of ML in the diagnosis of dementia and neurodegenerative diseases.

## 2. Literature Review

In 1984, a report by the National Institutes of Neurological and Communicative Disorders and Stroke and the Alzheimer’s Disease and Related Disorders Association highlighted an increase in slow-wave band activity in EEGs of individuals with Alzheimer’s Disease (AD), suggesting EEG as a potential diagnostic tool [19]. Between 1985 and 1990, studies on AD EEG signals generally found increased low-frequency band power and decreased high-frequency band power [20,21].

EEG classification studies, along with the datasets used, frequency bands, feature extraction methods, classifiers employed, performance metrics, reported limitations, and the clinical significance of the results, are summarized in Table 1.

## 3. Materials and Methods

**Figure 1:**
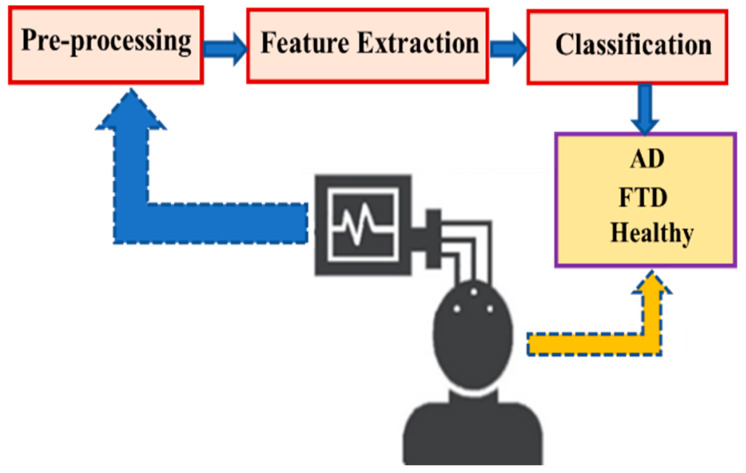
Representation of the designed system.

In this study, a publicly available EEG dataset was employed to support the diagnosis of Alzheimer’s Disease. The data analysis followed a structured pipeline comprising three main stages: pre-processing, feature extraction, and classification, as illustrated in Figure 1. Each stage of this pipeline is described in detail in the subsequent sections to ensure transparency and reproducibility of the methodology.

### 3.1. Dataset

This study used an open access EEG dataset recorded by the neurology team of the second Neurology Department at Thessaloniki AHEPA General Hospital [30]. This dataset consists of EEG signals recorded from subjects resting with closed eyes. The dataset includes 36 AD patients, 23 FTD patients, and 29 HC. The EEG data of 36 AD patients, 23 FTD patients, and 29 HC were processed in the study.

The neurological and cognitive status of the subjects was assessed using the International Mini-Mental State Examination (MMSE). The MMSE score ranges from 0 to 30, with lower scores indicating severe cognitive decline. The average MMSE score for AD subjects was 17.75, with a standard deviation of 4.5, while the average MMSE score for FTD subjects was 22.17, with a standard deviation of 8.22. The MMSE score for healthy subjects was reported to be 30. The median duration of the disease among the subjects was 25 months. Table 2 presents the demographic characteristics of AD, FTD, and HC.

**Figure 2:**
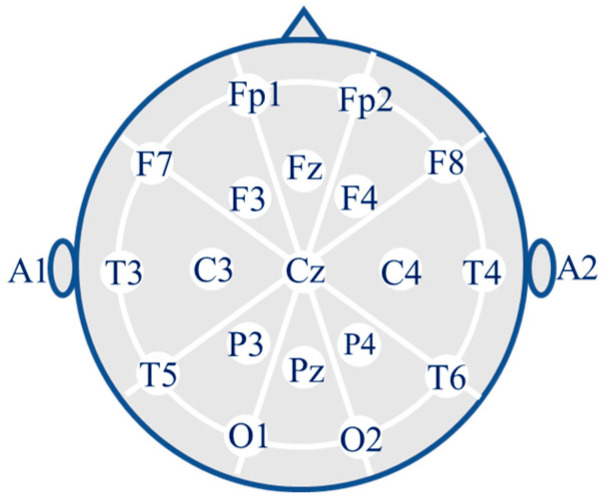
Placement of the 19 electrodes.

The EEG signals of the subjects were recorded using 19 scalp electrodes (Fp1, Fp2, F7, F3, Fz, F4, F8, T3, C3, Cz, C4, T4, T5, P3, Pz, P4, T6, O1, and O2) and two reference electrodes (A1 and A2) from the Nihon Kohden EEG 2100 clinical device as shown Figure 2. According to the international 10–20 electrode placement system, the electrodes were placed on the scalp. The A1 and A2 reference electrodes were used for impedance control. Before each recording, the skin impedance was adjusted to be below 5 kΩ. The sampling rate of the recordings was 500 Hz with a resolution of 10 μV/mm. Table 3 provides detailed information about the recording parameters of the dataset.

During the acquisition of EEG signals, artifacts from the environment and the subject’s own physiological movements can be introduced into the EEG signal. These artifacts can cause distortions in the EEG signal. To obtain reliable features, the signal is cleaned of artifacts before or after the ADC process using filters [12]. Artifact removal is critical for the effective processing of EEG signals.

This study used EEG recordings that had been pre-processed and cleaned of artifacts. The researchers who prepared the dataset initially applied a 0.5–45 Hz Butterworth band pass filter and re-referenced the signals to the A1-A2 channels. Subsequently, Artifact Subspace Reconstruction (ASR) was applied using EEGLAB to remove system artifacts from the signal. ASR is a method where unwanted high-variance channel data is removed, and the channel is reconstructed from the remaining data. Clean data portions are automatically identified, and unwanted segments are removed by setting a threshold [31]. During ASR, the maximum acceptable duration was set to 0.5 and the window standard deviation to 17. Following this, Independent Component Analysis (ICA) with the RunICA algorithm was applied to EEGLAB, and data of physiological artifacts were cleaned as much as possible using the “ICLabel” classification. ICA is based on the principle that signals can be separated into independent components. It separates and cleans artifacts from signals [12,32].

### 3.2. Pre-Processing

**Figure 3:**
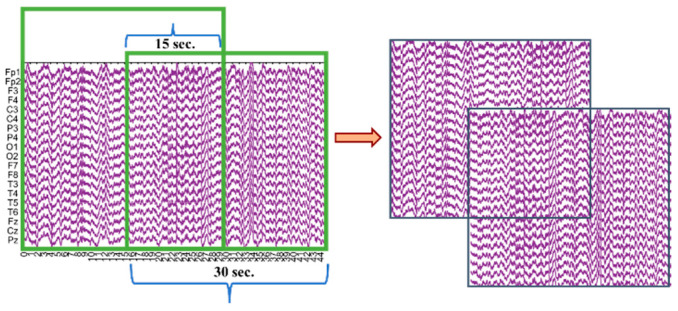
Illustration of the EEG signal segmentation into 30 s epochs with 50% overlap.

In addition to the pre-processing steps performed in the study, EEG recordings of different durations were segmented into epochs. The literature review shows that EEG recordings can be divided into epochs ranging from 2 to 30 s and can include overlap [10,27,33]. Based on this, the EEG recordings were segmented into 30 s epochs with 50% overlap, as shown in Figure 3. Segmenting the recordings with overlap minimized data loss and increased the number of data segments available for processing.

### 3.3. Feature Extraction

The primary goal of feature extraction is to identify distinctive and meaningful features from pre-processed signals and to create a feature vector. It is intended to perform feature extraction with reduced data rather than the full dataset, which can improve classification performance. Creating a feature vector reduces the data, increasing the classification’s training speed and enhancing the model’s accuracy [7].

Distinctive and meaningful features of the signals can appear in the time, frequency, and time–frequency domains. In the time domain, features such as statistical measures and Hjorth parameters of the signal are obtained [34]. When the features in the time domain are insufficient for analyzing and classifying the signal, the signal is transformed into the frequency or time–frequency domain, where additional features are explored.

This study performed feature extraction on the pre-processed signals using both the time and spectral domains. Each channel of the EEG signal was converted from the time domain to the spectral domain using Welch’s spectral analysis in the 0.5–45 Hz range. The spectral domain is a commonly used method for distinguishing signals and extracting information from relevant data [35].

Welch’s spectral analysis divides the input signal into overlapping segments. A chosen window function is applied to each segment. The Fast Fourier Transform (FFT) is applied to the windowed segments to compute the periodogram of each segment, and the average periodogram of the windowed segments is calculated as shown in Equation (1) [35].

(1) $$S\left(w\right)=\frac{1}{L}\sum_{l=1}^{L}\emptyset_{l}\left(w\right)$$

where L is the total number of windowed segments, $\emptyset_{l}\left(w\right)$ is the periodogram of the windowed segments, and S(w) is the average periodogram.

In this study, the “Hamming” window function was chosen, and the overlap rate was set to 75% [33] to obtain the PSD of the signal.

For each of the 19 channels of the EEG signal, 7 features were identified in the time domain and 11 features in the spectral domain. A total of 18 features were extracted for each channel. The identified features determined in the time and spectral domains have been calculated using the formulas in Table 4.

Spectral-domain feature extraction was performed using frequency ranges for 5 sub-bands, as shown in Table 5. The power of these frequency ranges for the defined sub-bands was calculated using the BP formula in Equation (9) in Table 4. The ratios between the bands were obtained in Equation (10) in Table 4.

Before proceeding to the feature selection phase, the feature vectors were divided into two sets using systematic sampling: 70% for training and 30% for testing.

### 3.4. Feature Selection

A feature vector consisting of a total of 342 features (19 channels × 18 features per channel) was created from the EEG signals. High-dimensional feature vectors can contain irrelevant or redundant data, which may prolong and complicate the learning process during classification. Feature selection is performed to speed up the learning process and improve class discrimination. Principal Component Analysis (PCA), Independent Component Analysis (ICA), Correlation Coefficient, and Conditional Mutual Information Maximization (CMIM) are some approaches used for feature selection [36].

In this study, the Correlation Coefficient approach was applied for feature selection. In this approach, the relationship of a single feature with the label is examined to determine how well the feature contributes to class separation, and the features are ranked based on their contribution [36]. Two different correlation coefficient approaches, Spearman and Pearson, were used to select the best correlation coefficient approach. The average of the correlation coefficients was used to set a threshold, and features exceeding this threshold were used to create new feature vectors.

Spearman’s correlation coefficient is calculated as shown in Equation (11) [37], and Pearson’s correlation coefficient is calculated as shown in Equation (12) [38]:

(11) $$r_{s}=1-\frac{6\sum d_{i}^{2}}{N\left(N^{2}-1\right)}$$

where $r_{s}$ is Spearman’s correlation coefficient, $d_{i}$ is the difference between each pair of ranked variables, and N is the total number of samples.

(12) $$p\left(x,y\right)=\frac{\sum \left[xy\right]}{\sigma_{x}\sigma_{y}}$$

where p(x,y) is the Pearson correlation coefficient between variables *x* and y, $\sum \left[xy\right]$ is the cross-correlation between *x* and *y*, and $\sigma_{x}$ and $\sigma_{y}$ are the variances of *x* and *y* signals, respectively.

Since correlation coefficients with negative values indicate an inverse relationship, the absolute values of these coefficients were taken, and the threshold $R_{TH}$ value was calculated for both correlation coefficient approaches as given in Equation (13).

(13) $$R_{TH}=\frac{1}{K}\sum_{k=1}^{K}r_{k}$$

where $R_{TH}$ is the average of the correlation coefficients, K is the number of features, and $r_{k}$ is the correlation coefficient for the k-th feature.

The feature selection was performed using the approach that provided the best threshold value from Pearson and Spearman correlation coefficients, resulting in a new feature vector. The study used three feature vectors: one with all features without selection and the others are vectors containing more meaningful features selected after the feature selection process. In order to visualize these three high-dimensional datasets, t-Distributed Stochastic Neighbor Embedding (t-SNE) [39] was used to visualize the datasets.

### 3.5. Hyperparameter Optimization

Hyperparameter optimization aims to determine the optimal parameter combinations of the classification algorithms to be used prior to the training phase, in order to achieve the best training performance. Hyperparameters can be determined in two main ways: manual search or automated search. In manual search, the parameters of the chosen classification algorithm are adjusted one by one to reach the best possible performance, which is often time-consuming and computationally expensive. In contrast, automated search involves a systematic exploration of the parameter space to identify the combination that yields the best performance [40]. In this study, we employed a Gaussian Process (GP) Bayesian optimization method [41,42], which is widely used for optimizing objective functions that are costly to evaluate. The mathematical expressions used in Bayesian optimization are presented below.

(14) $$x^{*}=\arg{min}_{x\in H}f(x)$$

(15) $$f\left(x\right)\sim GP(\mu \left(x\right),\sigma^{2}\left(x\right))$$

(16) $$EI\left(x\right)=E\left[max(0,f\left(x_{best}\right)-f\left(x\right))\right]$$

(17) $$P\left(f|D_{new}\right)=\frac{P\left(D_{new}|f\right)P\left(f|D_{old}\right)}{P(D_{new})}$$

where $x^{*}$ denotes the point that minimizes the objective function, while H defines the search space over which the optimization is to be performed. The unknown function f(x) is approximated using a Gaussian Process defined by a mean function $\mu \left(x\right)$ and a variance $\sigma^{2}\left(x\right)$, which capture the expected value and the uncertainty of the prediction at each point, respectively. The term $f\left(x_{best}\right)$ refers to the best function value observed so far, and $EI\left(x\right)$ quantifies the expected improvement resulting from evaluating point x. The posterior distribution $P\left(f|D_{new}\right)$ is obtained by updating the prior $P\left(f|D_{old}\right)$ with new data $D_{new}$, using the likelihood $P\left(D_{new}|f\right)$. The term $P(D_{new})$ serves as a normalization constant, ensuring the posterior is a valid probability distribution.

### 3.6. Classification

ML, a subfield of artificial intelligence, can be defined as computer models and algorithms that automatically learn from data and experiences using mathematics, statistics, optimization, and knowledge discovery to solve tasks or problems [43]. ML can be categorized into supervised, unsupervised, reinforcement, and deep learning [44].

Supervised learning involves creating a model by using relationships between a predefined set of inputs and target outputs to train the system. Supervised learning algorithms are divided into classification and regression [43].

Classification is one of the supervised learning algorithms in ML. It involves labeling data to determine which class it belongs to, using an algorithm to train the feature vectors allocated for training, and then deciding which class an unknown feature vector belongs to through a decision mechanism [45].

This study used SVM and k-NN classification algorithms to diagnose AD using EEG signal feature vectors. SVM is a powerful ML model based on kernels. SVM is a learning algorithm aimed at classifying data by finding the optimal hyperplane in a space called the feature space, which is formed from the training data [46]. The position and orientation of the hyperplane are adjusted to achieve the best classification [47]. When the data in the feature space is not linearly separable, the feature space is transformed into a higher-dimensional space using the “kernel trick” to classify the data [48]. The quadratic kernel function used in this study is calculated as shown in Equation (18) [49]:

(18) $$Q\left(x_{i}x_{j}\right)=\sqrt{{\left\|x_{i}-x_{j}\right\|}^{2}+c^{2}}$$

k-NN is a learning algorithm that aims to classify based on the distance to the nearest neighbors (k) in the feature space of the attributes [50]. Distance measurements are calculated according to distance metrics such as Euclidean, Cosine, Chebyshev, and Mahalanobis. The distance metric used in this study is cosine, calculated as shown in Equation (19) [51]:

(19) $$\cos\left(x,y\right)=1-\frac{\sum_{i=1}^{N}x_{i}y_{i}}{\sqrt{\sum_{i=1}^{N}x_{i}^{2}}\sqrt{\sum_{i=1}^{N}y_{i}^{2}}}$$

### 3.7. Performance Evaluation

A confusion matrix is a metric table used to understand and evaluate the performance of classification algorithms by calculating statistical measurements [52]. The confusion matrix visualizes the model’s actual and predicted classes and calculates performance metrics such as accuracy, recall, specificity, precision, and F1 score [53]. The structure of the confusion matrix is shown in Table 6. In the table, TP, FP, TN, and FN represent True Positives, False Positives, True Negatives, and False Negatives, respectively [54].

In this study, the metrics and formulas used to evaluate classification performance with the confusion matrix are provided in Table 7. Accuracy calculates the proportion of correct predictions over the data points assessed [55]. Recall/Sensitivity calculates the proportion of correctly classified true positive data [56]. Specificity calculates the proportion of correctly classified true negative data [55]. Precision calculates the proportion of truly positive examples among those predicted as positive [56]. Negative predictive value (NPV) indicates the proportion of truly negative cases among those predicted as negative by the model. False discovery rate (FDR) is the proportion of actually negative cases among the samples that the model predicted as positive [57]. Balanced Classification Rate (BCR) is the average of per-class sensitivity and specificity, providing a balanced measure for multi-class classification tasks [58]. The F1 Score is obtained by calculating the harmonic mean of recall and precision [55]. In addition to these metrics, the Receiver Operating Characteristic (ROC) curve is also used to measure performance. ROC curves are a technique used to evaluate and visualize the performance of classification algorithms. The ROC curve plots sensitivity (on the *y*-axis) against specificity (on the *x*-axis) at different points of the model. The area under the plotted curve is called the Area Under Curve (AUC). The AUC value ranges from 0 to 1, providing information about the classification’s performance. An AUC value of 1 indicates better classification performance [59].

## 4. Results and Discussion

This study’s EEG signals obtained from patients with AD, FTD, and HC were processed through pre-processing and feature extraction stages and classified using ML methods, specifically the SVM and k-NN algorithms. The aim was to detect whether the EEG signals in the test data, which were separated and included in the system through systematic sampling, indicated the presence of Alzheimer’s Disease. This section presents the methods used and their classification results, evaluating the performance rates of the classification algorithms.

All stages of the study were conducted using MATLAB R2021b. In the initial stage, EEG signals obtained from the dataset were pre-processed and segmented into 30 s epochs with 15 s overlaps. The resulting number of epochs for AD, FTD, and HC is 1888, 1074, and 1563, respectively.

After the pre-processing stage, features were extracted from EEG signals both in the time and spectral domains. Welch spectral analysis was used to obtain the PSD of the EEG signals, and a transition to the spectral domain was performed.

During feature extraction, seven features were extracted from the time domain for each channel of the EEG signal. In the spectral domain, the signal was divided into five frequency bands, and each band’s power and the bands’ ratios other than the gamma band were calculated. Since AD primarily affects low-frequency wave activities, the ratio of the gamma band to the other bands was not considered. For the spectral domain, 11 features were extracted from each channel of the EEG signal.

For each 30 s EEG signal from 19 channels, 342 features (19 channels × 18 features) were extracted, resulting in a feature vector of size 4521 × 342.

The feature vectors, consisting of 342 features, were divided into training and test datasets, 70% of which were used for training and 30% for testing through systematic sampling. The feature vectors are divided into training and test datasets to determine how well the system classifies previously unseen test data. Systematic sampling was used to ensure that data from a particular group did not dominate either the training or the test datasets. Systematic sampling was employed to achieve an equal distribution. Table 8 shows that the number of samples in the AD group is higher than in the FTD and HC. This is because the AD group has more EEG recordings and longer recording durations than the FTD and HC groups. Percentile distributions were adjusted based on these values through systematic sampling.

To evaluate the effectiveness and discriminatory power of the classification, the first 18 features extracted from the records of AD, HC, and FTD individuals (data numbers 1, 1889, and 3452, respectively), and the Spearman and Pearson correlation coefficients for these features are presented in Table 9. Spearman and Pearson correlation coefficients were calculated only for the training dataset in order to prevent data leakage.

To determine the features with the best correlation with the labels, the absolute values of the negative correlation coefficients were taken, and the $R_{TH}$ value was calculated for both correlation methods. For the Spearman correlation coefficient method, $R_{TH\_spearman}=0.1398$ was computed. For the Pearson correlation coefficient method, $R_{TH\_pearson}=0.0969$ was computed.

When both correlation methods were examined, it was found that the Spearman correlation coefficient method had a better threshold value. While there were 130 features above the threshold value in the Spearman correlation coefficient method, there were 137 features above the threshold value in the Pearson correlation coefficient method. Based on these results, feature selection was performed using the Spearman correlation coefficient. A new, smaller feature vector was created with the 130 more meaningful features above the $R_{TH\_spearman}$ value. The 130 features selected according to the Spearman correlation coefficient approach are listed in Table 10.

**Figure 4:**
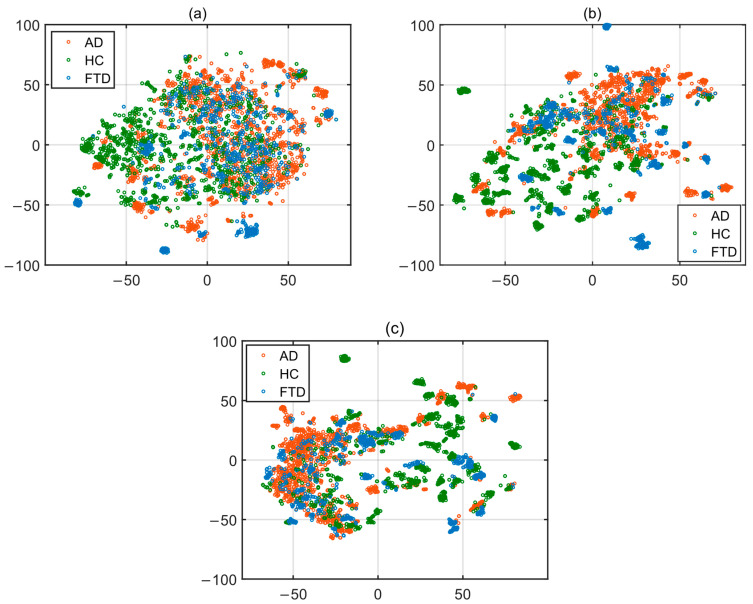
t-SNE 2-D embedding for training set: (**a**) all (342) features; (**b**) 130 features; (**c**) 50 features.

When examining the features selected using the Spearman correlation coefficient method, the feature with the highest correlation to the label is feature number 268, Pz/TABPR. The Pz/TABPR feature represents the theta-alpha band power ratio in the parietal region of the brain, with a correlation coefficient of 0.5259. All of the 130 selected features were derived from the spectral domain. This result demonstrates the importance of feature extraction in the spectral domain for the classification stage. A new training set was created by selecting the top 50 features out of the 130 selected features. These three high-dimensional datasets were visualized by applying t-SNE in two-dimensional form and are shown in Figure 4.

For the classification using the SVM and k-NN algorithms, the best training accuracy rates were achieved by determining some hyperparameters of the algorithms through Bayesian optimization.

In the SVM classification algorithm, after setting the kernel function to “quadratic,” the kernel scale to “automatic”, the box constraint levels were determined by the Bayesian optimization method. As a result of this process, the box constraint value and classification accuracy were found as follows: 2.4972 and 90.81% for the training dataset with 342 features, 3.9812 and 92.60% for the training dataset with 130 features, and 5.5952 and 88.06% for the training dataset with 50 features. The analysis of different parameter values was conducted, and a portion of the results from this analysis is provided in Table 11.

In the k-NN classification algorithm, after selecting the distance metric as “cosine” and the distance weight as “squared inverse,” the number of neighbors (k) was determined using the Bayesian optimization method. As a result of this process, the k value and classification accuracy were found as follows: k = 6 and 77.88% accuracy for the training dataset with 342 features, k = 2 and 90.05% accuracy for the training dataset with 130 features, and k = 4 and 89.03% accuracy for the training dataset with 50 features. A portion of the analysis of the k-NN algorithm parameters is provided in Table 12.

**Figure 5:**
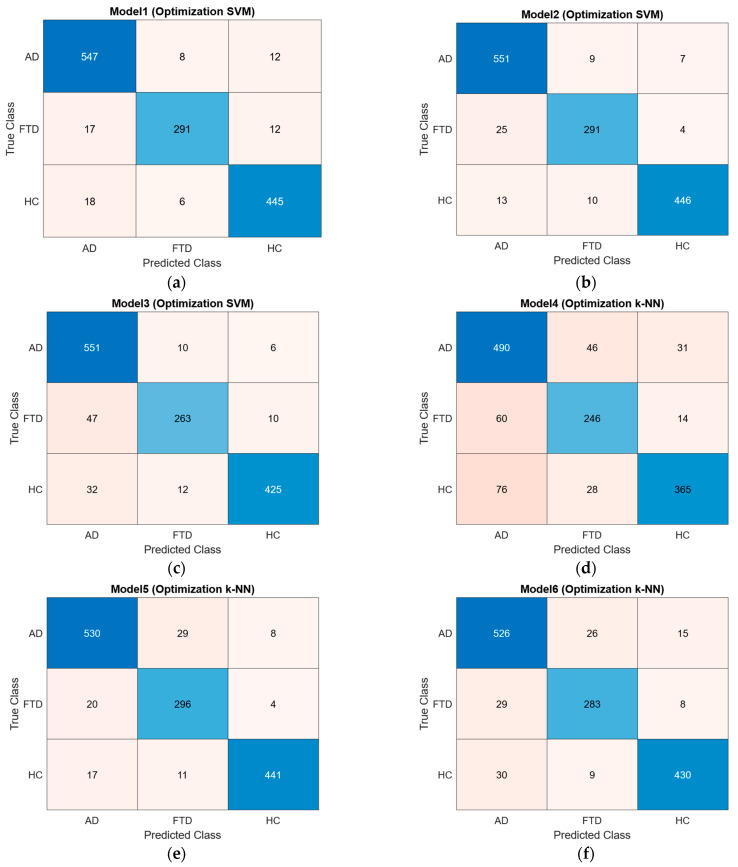
Confusion matrices: (**a**) SVM model trained with 342 features; (**b**) SVM model trained with 130 features; (**c**) SVM model trained with 50 features; (**d**) k-NN model trained with 342 features; (**e**) k-NN model trained with 130 features; (**f**) k-NN model trained with 50 features.

**Figure 6:**
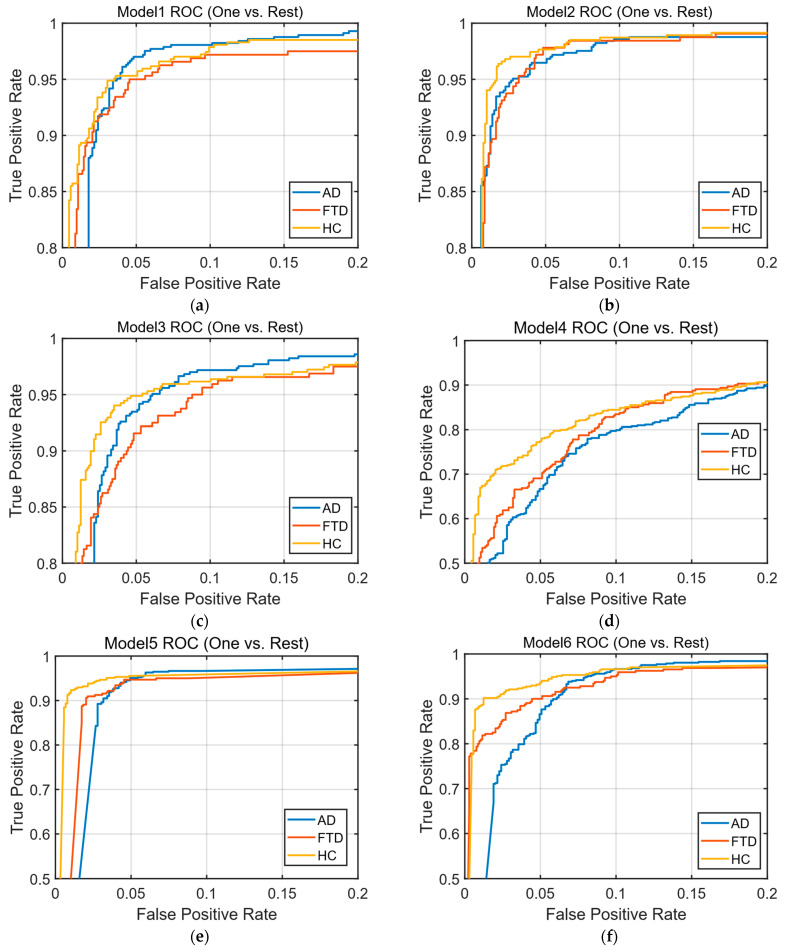
ROC (one-vs.-rest) curves: (**a**) ROC curve of the SVM model trained with 342 features; (**b**) ROC curve of the SVM model trained with 130 features; (**c**) ROC curve of the SVM model trained with 50 features; (**d**) ROC curve of the k-NN model trained with 342 features; (**e**) ROC curve of the k-NN model trained with 130 features; (**f**) ROC curve of the k-NN model trained with 50 features.

Based on the parameters determined, training datasets consisting of 342, 130, and 50 features were trained using the SVM and k-NN classification algorithms. After training, the classification algorithms were tested using a test dataset that the system had not previously encountered, resulting in confusion matrices and ROC curves. The obtained confusion matrices are presented in Figure 5, and the ROC curves are shown in Figure 6.

**Figure 7:**
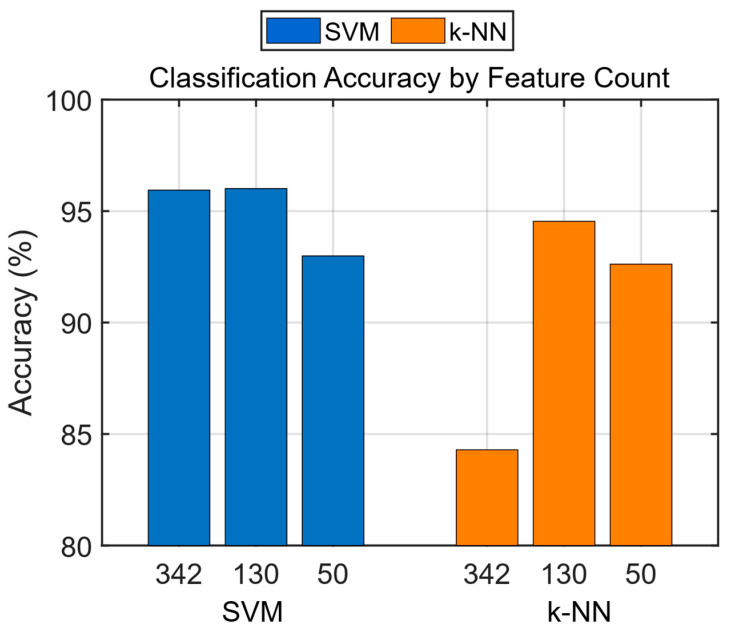
Classification accuracy by feature count.

The accuracy, sensitivity, specificity, precision, NPV, FDR, BCR, F1 Score, and AUC values were calculated from the confusion matrices of the trained models. As shown in Table 13, the best classification accuracy performance was achieved with the SVM algorithm using the feature vector of 130 features, with an accuracy of 96.01%. When comparing accuracy performances, models trained with 130 features outperformed those trained with 342 features and 50 features. The obtained accuracy rates are presented as a bar chart in Figure 7.

When the studies conducted on the dataset used in this study are examined, the metrics and performance results for Alzheimer’s disease (AD) diagnosis from EEG signals are presented in Table 14**.**

While diagnosing AD from EEG signals, various parameters and methods—such as the feature extraction techniques, signal ranges, and classification algorithms—directly influence the system’s performance.

In light of this information, it is observed that the proposed system achieves better accuracy performance compared to the other studies.

## 5. Conclusions

This study introduces a machine learning-based approach for diagnosing AD using EEG signals, focusing on enhancing accuracy and efficiency in the diagnostic process. The system utilized SVM and k-NN classifiers, with features extracted from the time and spectral domains. The selection of 130 highly discriminative features, predominantly derived from spectral analysis, was crucial in improving classification accuracy.

The SVM algorithm, particularly when applied to the reduced feature set, achieved a superior accuracy of 96.01% and it has outperformed other studies that used the same dataset. This significant improvement underscores the effectiveness of the feature selection process and the optimization of classifier parameters through Bayesian optimization.

The results of this study demonstrate the potential of machine learning to automate and expedite the AD diagnostic process, offering a valuable tool to complement traditional methods. However, despite these promising outcomes, achieving the highest possible accuracy remains essential, especially given the profound impact of AD on individuals and their families.

Despite the promising results obtained in this study, several limitations should be taken into account. Firstly, the dataset was not split on a subject-wise basis, which may have led to data leakage. Additionally, the use of only a single dataset limits the generalizability of the proposed approach. The study employed a limited number of feature selection methods, and the absence of alternative techniques such as ReliefF and statistical approaches may have resulted in overlooking more appropriate feature subsets. Furthermore, only two classification algorithms (SVM and k-NN) were used, and no comparisons were made with advanced techniques such as deep learning or ensemble methods. Lastly, no analysis was performed to identify which EEG channels contributed most significantly to classification performance; however, such channel-based analyses could enhance the diagnosis of Alzheimer’s disease.

In future work, we aim to address the limitations of the current study by enhancing the feature extraction process and investigating alternative feature selection techniques to further improve classification performance. Additionally, we plan to explore advanced classification methods, including ensemble learning algorithms and deep neural network architectures, and systematically compare their performance with traditional machine learning models. These advancements are expected to contribute to the development of a more robust and generalizable framework for EEG-based Alzheimer’s Disease diagnosis. Ultimately, such efforts could lead to improved diagnostic accuracy, earlier detection, and better patient care.

## Figures and Tables

**Table 1:** Literature review.

Author(s), Year	Dataset	Bands Used	Feature Extraction	Classifiers Applied	Metrics & Performance	Limitations	Significance
Lehmann et al., 2007 [22]	116 mild AD, 81 moderate AD, 45 HC	Delta, Theta, Alpha1, Alpha2, Beta1–3	Spectral power, centroids, synchronization (hand-crafted)	PC-LDA, PLS-LDA, PC-LR, PLS-LR, Bagging, RF, SVM, NN	SVM and NN (Mod. AD vs. HC: Sens. 89%, Spec. 88%)	High sensitivity to feature selection, sample imbalance risk.	Demonstrated feasibility of EEG-based AD classification; modern ML methods are slightly superior.
Sankari and Adeli, 2011 [23]	20 AD, 7 HC	Delta, Theta, Alpha, Beta	Coherence and wavelet coherence (hand-crafted)	PNN	Conventional coherence: 100% accuracy	Small sample size, potential overfitting.	Demonstrated potential of coherence measures and PNN in early AD diagnosis using EEG.
Morabito et al., 2016 [24]	63 AD, 56 MCI, 23 HC	0.1–30 Hz total (includes Delta, Theta, Alpha, Beta)	CWT + time–frequency stats; CNN learns latent features; mix of hand-crafted and automatic	CNN	AD/MCI/HC: 82% acc., 83% sens., 75% spec.	Better training accuracy (95%).	Deep CNN effectively extracted latent EEG features.
Fiscon et al., 2018 [25]	49 AD, 37 MCI, 23 HC	Delta, Theta, Alpha, Beta, Gamma	FFT, DWT (hand-crafted)	DT	AD vs. HC: 83% acc.	Small sample, limited generalizability.	Shows strong potential for early AD detection.
Bairagi et al., 2018 [26]	20AD, 25HC	Delta, Theta, Alpha, Beta	DWT (hand-crafted)	SVM, k-NN	94% acc.	Small dataset; limited generalizability.	Combining entropy and fractal features with wavelet analysis yields high accuracy for AD detection.
Durongbhan et al., 2019 [3]	20 AD, 20 HC	Delta, Theta, Alpha, Beta	FFT, CWT (hand-crafted);	k-NN, SVM, DT	k-NN: FFT features: 97% acc., CWT features: 99% acc.	Relatively small dataset, class balance and overfitting risk not detailed.	Spectral features from FFT and CWT combined with k-NN yielded high AD classification accuracy.
Vecchio et al., 2020 [27]	175 AD, 120 HC	Delta, Theta Alpha1, Alpha2, Beta 1, Beta2, Gamma	Lagged Linear Coherence (hand-crafted)	SVM	95% ± 3% acc.	Limited to logistic regression, no external validation.	LLC features can effectively classify AD vs. HC with high accuracy.
Safi & Safi, 2021 [1]	EEG; 31 mild AD, 20 moderate AD, 35 HC	Delta, Theta, Alpha, Beta	PSD, DWT, EMD (hand-crafted)	k-NN, SVM, RLDA	97.64% acc.	Performance varies across decomposition methods.	Demonstrated effectiveness of decomposition-based Hjorth features in classifying AD severity.
AlSharabi et al., 2022 [7]	EEG; 31 mild AD, 22 moderate AD, 35 HC;	Delta, Theta, Alpha, Beta, Gamma	DWT + statistical features (hand-crafted)	LDA, QDA, SVM, k-NN, NB, DT, ELM, ANN, RF	99.98% acc. with k-NN using DWT features	No external validation, dataset overlap risk.	Very high accuracy using DWT-based features suggests strong discriminative potential for AD stages.
Göker et al., 2023 [28]	24 AH, 24 HC	Delta, Theta, Alpha, Beta, Gamma	PSD (hand-crafted)	SVM, k-NN, RF, BiLSTM	98.85% acc. for the HC class	Relatively small dataset, no external validation.	In the EEG dataset, channels from individual subjects were treated as independent recordings.
Kim et al., 2025 [29]	20 SCD, 28 MCI, 10 AD	Delta, Theta, Alpha, Beta, Gamma	Automatic feature extraction	EEG Conformer/ Attention-LSTM	Resting-state acc: 71.67%, Tasking-state acc: 79.16%	AD-MCI separation remains challenging.	Shows promise in differentiating SCD, MCI, and AD using EEG-based deep learning.

**Table 2:** Demographic information of the dataset.

	Gender	Mean Age
AD	13 Males/23 Females	66.4 (±7.9)
FTD	14 Males/9 Females	63.6 (±8.2)
HC	11 Males/18 Females	67.9 (±5.4)

**Table 3:** Record information on the dataset.

	Recording Time (Minute)		
	Minimum	Maximum	Total
AD	5.1	21.3	485.5
FTD	7.9	16.9	276.5
HC	12.5	16.5	402

**Table 4:** Formulas for features determined in the time domain and spectral domain.

Feature Number	Domain	Feature	Equation	Equation Number
1	Time	Kurtosis (KU)	$\sum_{n=1}^{N}\left(x(n\right)-AVG)\frac{4}{\left(N-1\right){SD}^{4}}$	(2)
2		Average (AVG)	$\frac{1}{N}\sum_{n=1}^{N}x\left(n\right)$	(3)
3		Root Mean Square (RMS)	$\sqrt{\frac{\sum_{n=1}^{N}{\left|x\left(n\right)\right|}^{2}}{N}}$	(4)
4		Skewness (SK)	$\sum_{n=1}^{N}\left(x(n\right)-AVG)\frac{3}{\left(N-1\right){SD}^{3}}$	(5)
5		Standard deviation (SD)	$\sqrt{\frac{1}{N}\sum_{n=1}^{N}{\left(x\left(n\right)-AVG\right)}^{2}}$	(6)
6		Variance (VAR)	$\frac{1}{N}\sum_{n=1}^{N}{\left(x\left(n\right)-AVG\right)}^{2}$	(7)
7		Norm (NOR)	$\sqrt{\sum_{n=1}^{N}{\left|x(n)\right|}^{2}}$	(8)
8	Spectral	Delta Band Power (DBP)	$\frac{1}{N}\sum_{i}^{N}\left|X_{i}^{2}\right|$	(9)
9		Theta Band Power (TBP)		
10		Alpha Band Power (ABP)		
11		Beta Band Power (BBP)		
12		Gamma Band Power (GBP)		
13		Delta-Theta Band Power Ratio (DTBPR)		
14		Delta-Alpha Band Power Ratio (DABPR)	$\frac{P_{x}}{P_{y}}$	(10)
15		Delta-Beta Band Power Ratio (DTBPR)		
16		Theta-Alpha Band Power Ratio (TABPR)		
17		Theta-Beta Band Power Ratio (TBBPR)		
18		Alpha-Beta Band Power Ratio (ABBPR)		

**Table 5:** Sub-band frequency ranges.

	Frequency Range
Delta	0.5–4 Hz
Theta	4–8 Hz
Alpha	8–13 Hz
Beta	13–25 Hz
Gamma	25–45 Hz

**Table 6:** Confusion matrix.

		Predicted	
		Positive	Negative
True	Positive	TP	FN
	Negative	FP	TN

**Table 7:** Metrics used for performance evaluation.

Feature	Equation	Equation Number
Accuracy	$\begin{array}{c} \frac{TP+TN}{TP+FP+TN+FN}\times 100 \end{array}$	(20)
Sensitivity	$\begin{array}{c} \frac{TP}{TP+FN} \end{array}$	(21)
Specificity	$\begin{array}{c} \frac{TN}{TN+FP} \end{array}$	(22)
Precision	$\begin{array}{c} \frac{TP}{TP+FP} \end{array}$	(23)
NPV	$\frac{TN}{FN+TN}$	(24)
FDR	1-Precision	(25)
BCR	$\frac{1}{C}\sum_{i=1}^{C}\left(\frac{{sensitivity}_{i}+{specificity}_{i}}{2}\right)$	(26)
F1 Score	$\frac{2\times Sensitivity\times Precision}{Sensitivity+Precision}$	(27)

**Table 8:** Dataset distributions.

Dataset	Label	Data Count	Proportion	Total Data Count	Overall Ratio
Training	AD	1321	41.74%	3165	70%
	FTD	750	23.70%		
	HC	1094	34.57%		
Testing	AD	567	41.81%	1356	30%
	FTD	320	23.60%		
	HC	469	34.59%		

**Table 9:** Results of feature extraction and feature–label relationships based on Spearman and Pearson correlation coefficients.

No	Feature	Data Number			Spearman Corr. Coeff.	Pearson Corr. Coeff.
		1	1189	3452		
		Label				
		AD	HC	FTD		
1	Fp1/KU	2.9388	3.7483	2.7580	−0.0016	0.0111
2	Fp1/AVG	0.5395	0.0301	−0.6613	−0.0258	0.0251
3	Fp1/RMS	31.3199	36.5374	32.3603	0.0383	0.0451
4	Fp1/SK	0.1353	0.5760	0.1422	0.0361	0.0266
5	Fp1/SS	31.3163	36.5386	32.3546	0.0382	0.0447
6	Fp1/VAR	980.7131	1335.0740	1046.8250	0.0382	0.0415
7	Fp1/NO	3835.8940	4474.9080	3963.3200	0.0383	0.0451
8	Fp1/DBP	398.0764	617.8834	668.4050	−0.0224	−0.0444
9	Fp1/TBP	53.1110	43.7246	54.8660	−0.2247	−0.1329
10	Fp1/ABP	13.0952	26.2170	20.6642	0.2161	0.2019
11	Fp1/BBP	15.7033	11.5111	9.4213	0.0866	0.0487
12	Fp1/GBP	22.1014	5.0517	3.8901	−0.0849	−0.0089
13	Fp1/DTBPR	0.1334	0.0707	0.0820	−0.2062	−0.1416
14	Fp1/DABPR	0.0328	0.0424	0.0309	0.2393	0.2055
15	Fp1/DBBPR	0.0394	0.0186	0.0140	0.0983	0.0573
16	Fp1/TABPR	0.2465	0.5995	0.3766	0.4279	0.2785
17	Fp1/TBBPR	0.2956	0.2632	0.1717	0.2610	0.0969
18	Fp1/ABBPR	1.1991	0.4390	0.4559	−0.1956	−0.0893

**Table 10:** The 130 features were selected according to the Spearman correlation coefficient approach.

Selected Features
268, 178, 286, 160, 124, 176, 172, 269, 266, 262, 232, 179, 340, 125, 16, 233, 34, 142, 287, 304, 158, 214, 263, 154, 280, 180, 284, 70, 161, 267, 173, 52, 118, 122, 250, 162, 196, 177, 341, 119, 143, 270, 305, 338, 334, 288, 140, 281, 136, 155, 123, 159, 189, 88, 285, 227, 106, 335, 231, 342, 251, 230, 144, 126, 89, 107, 225, 137, 226, 17, 36, 339, 197, 297, 141, 32, 53, 193, 14, 45, 207, 322, 49, 243, 215, 302, 9, 28, 301, 212, 229, 27, 10, 83, 248, 63, 216, 244, 298, 208, 13, 82, 101, 35, 68, 71, 67, 247, 18, 64, 211, 72, 31, 100, 50, 174, 86, 306, 135, 46, 194, 104, 279, 299, 283, 245, 316, 87, 105, 303

**Table 11:** Partial analysis of SVM parameters using different training sets.

Kernel Function	Kernel Scale	Number of Features	Box Constraint Level	Training %
Quadratic	Automatic	342	1	89.66
			2.4972	90.81
			3	89.98
			4	89.98
			5	90.58
		130	1	92.13
			2	93.23
			3	93.17
			3.9812	92.60
			5	92.92
		50	2	87.42
			3	87.29
			4	87.64
			5	87.14
			5.5952	88.06

**Table 12:** Partial analysis of k-NN parameters using different training sets.

Distance Metric	Distance Weight	Number of Features	k	Training %
Cosine	Squared Inverse	342	2	76.87
			3	77.21
			4	78.60
			5	77.34
			6	77.88
		130	2	90.05
			3	89.92
			4	89.73
			5	89.85
			6	89.98
		50	2	88.65
			3	88.49
			4	88.69
			5	89.03
			6	88.53

**Table 13:** Performance metrics of the models: accuracy, sensitivity, specificity, precision, NPV, FDR, BCR, F1 Score, AUC.

Classification	Number of Features	Accuracy%	Sensitivity	Specificity	Precision	NPV	FDR	BCR	F1 Score	AUC
SVM	342	95.94	0.96	0.95	0.93	0.97	0.06	0.96	0.95	0.98
	130	96.01	0.97	0.95	0.93	0.97	0.06	0.96	0.95	0.98
	50	92.99	0.97	0.89	0.87	0.97	0.12	0.93	0.92	0.98
k-NN	342	84.29	0.86	0.82	0.78	0.89	0.21	0.85	0.82	0.93
	130	94.54	0.93	0.95	0.93	0.95	0.06	0.95	0.93	0.96
	50	92.62	0.92	0.92	0.89	0.94	0.10	0.93	0.91	0.97

**Table 14:** Comparison of the methodology of studies that use the same dataset as the dataset used in this study.

Ref.	Year	Band Power	Feature Extraction	Classifiers Applied	Metric and Performance
Miltidaous et al. [10]	2023	Delta, Theta, Alpha Beta, Gamma	Relative Band Power, Spectral Coherence Connectivity (SCC)	DICE-net	Acc: AD/HC = 83.23%
Wang et al. [60]	2023	Theta, Alpha Beta	PSD	SVM	AUC: AD/FTD = 0.73
Chen et al. [61]	2023	Delta, Theta, Alpha Beta, Gamma	CNN+ Visual Transformers (ViTs)	CNN	Acc: AD/FTD/HC = 80.23%
Velichko et al. [62]	2023	Delta, Theta, Alpha Beta, Gamma	New entropy: Neural Network Entropy (NNetEn)	SVM	Acc: AD/HC = 88.45%
Ma et al. [63]	2024	Delta, Theta, Alpha Beta, Gamma	PHI values for each electrode pair	SVM	Acc: AD/HC = 76.9%, FTD/HC = 90.4%
Rostamikia et al. [64]	2024	Delta, Theta, Alpha Beta, Gamma	Time and DWT	SVM	Acc: AD/FTD = 87.8%, AD + FTD/HC = 93.5%
Stefanou et al. [65]	2025	Delta, Theta, Alpha Beta, Gamma	FFT-based spectrograms	CNN	Acc: AD/HC = 79.45%, AD + FTD/HC = 80.69%
Proposed	2025	Delta, Theta, Alpha Beta, Gamma	Time and PSD	SVM	Acc: AD/FTD/HC = 96.01%

## Data Availability

The data supporting this study’s findings are available from the corresponding author upon reasonable request.

## Abbreviations

The following abbreviations are used in this manuscript:

AD	Alzheimer’s Disease
AUC	Area Under Curve
BILSTM	Bidirectional Long Short-Term Memory
BCR	Balanced Classification Rate
CC	Conventional Coherence
CNN	Convolutional Neural Networks
CWT	Continuous Wavelet Transform
DT	Decision Trees
DWT	Discrete Wavelet Transform
ELM	Extreme Learning Machine
EMD	Empirical Mode Decomposition
FDR	False Discovery Rate
FFT	Fast Fourier Transform
FTD	Frontotemporal Dementia
HC	Healthy Individuals
ICA	Independent Component Analysis
k-NN	k-Nearest Neighbors
ML	Machine Learning
MLP	Multilayer Perceptron Model
MMSE	Mini-Mental State Examination
NN	Neural Network
NPV	Negative Predictive Value
PCLDA	Principal Component Linear Discriminant Analysis
PCLR	Principal Component Logistic Regression
PLSLDA	Partial Least Squares LDA
PNN	Probabilistic Neural Network
PSD	Power Spectral Density
QDA	Quadratic Discriminant Analysis
RF	Random Forest
ROC	Receiver Operating Characteristic
SVMs	Support Vector Machines
t-SNE	t-distributed Stochastic Neighbor Embedding
WC	Wavelet coherence
